# Nitric oxide donor protects against acetic acid-induced gastric ulcer in rats via S-nitrosylation of TRPV1 on vagus nerve

**DOI:** 10.1038/s41598-017-02275-1

**Published:** 2017-05-18

**Authors:** Ting Han, Yan Tang, Jing Li, Bing Xue, Liping Gong, Jingxin Li, Xiao Yu, Chuanyong Liu

**Affiliations:** 1 Department of Physiology, School of Basic Medical Sciences, Shandong University Cheeloo Medical College, Shandong, China; 2 Provincial Key Lab of Mental Disorder, School of Basic Medical Sciences, Shandong University Cheeloo Medical College, Shandong, China

## Abstract

This study was conducted to investigate the effects of nitric oxide (NO) in acetic acid-induced gastric ulcer of rats and the underlying mechanisms. We found that peritoneal injection of sodium nitroprusside (SNP), a NO donor, decreased the ulcer area, inflammatory cell infiltration and MPO degree in acetic acid-induced gastric ulcer in rats. This effect was abolished by a transient receptor potential vanilloid 1 (TRPV1) antagonist or prior subdiaphragmatic vagotomy. SNP increased the jejunal mesenteric afferent discharge in a dose-depended manner, which was largely diminished by pretreatment of S-nitrosylation blocker N-ethylmaleimide, TRPV1 antagonist capsazepine, genetic deletion of TRPV1, or vagotomy. Whole-cell patch clamp recording showed that SNP depolarized the resting membrane potential of NG neurons, and enhanced capsaicin-induced inward current, which were both blocked by N-ethylmaleimide. Our results suggest that NO donor SNP alleviates acetic acid-induced gastric ulcer in rats via vagus nerve, while S-nitrosylation of TRPV1 may participate in this route. Our findings reveal a new mechanism for vagal afferent activation, and a new potential anti-inflammatory target.

## Introduction

Nitric oxide (NO), a free gas created from L-arginine and oxygen by nitric oxide synthases (NOS)^1^, is also a pleiotropic neurotransmitter within both the central and peripheral nervous system^2, 3^. Within the gastrointestinal (GI) tract, NO plays a critical role in regulating lots of physiological processes, including motility, mucus production and intestinal secretion, inflammatory responses, blood flow and vascular integrity^4, 5^. There are three isoforms of NOS: constitutive neuronal NOS (nNOS), constitutive endothelial NOS (eNOS), and inducible NOS (iNOS), which is induced during inflammation^6, 7^. NO is believed to mediate the cellular effects via the cyclic guanosine monophosphate (cGMP)-protein kinase G (PKG) pathway or cGMP-independent S-nitrosylation^8, 9^. The importance of S-nitrosylation modification towards protein thiols is increasingly recognized by researchers since it exerts mumerous ubiquitous influence of NO on cellular signal transduction^10, 11^. An increasing number of ion channels have been identified as targets for S-nitrosylation^8^.

Vagal afferents are extensively distributed in the GI tract to control and coordinate gastrointestinal functions, including the “sixth sense”^12^. Its effect in controlling the systemic and local inflammation has been well recognized. During systemic inflammation, vagal afferents are reported to monitor the increase of inflammatory cytokines, and the subsequent excitation of afferent fiber initiates a cholinergic anti-inflammation pathway. By activation of α7 nicotinic ACh receptors (nAChRs) on macrophage, this reflex results in inhibiting production of TNF-α and alleviating the inflammation^13, 14^. A similar vagally mediated anti-inflammatory reflex is reported to modulate inflammation in the GI tract, but the underlying mechanism, especially that of the modulation of the afferent discharge, has not been clearly understood^15–19^.

It has been demonstrated that NO excited vagus afferents in GI tract. NO donor activats gastric vagal afferents by mucosal NO-triggered 5-HT release in the rat stomach^20^. There is no convictive evidence to suggest that NO-induced vagal afferent firing evokes protective effect during inflammation. Our group has demonstrated that the iNOS inhibitor aminoguanidine increased the sensitivity of the vagus afferent to 5-HT mediated by LPS, while vagotomy reduced it^21, 22^. So, we hypothesize that NO might participate in the vagal anti-inflammatory reflex to modulate inflammatory responses.

In the present study, we examined the actions of NO on acetic acid-induced gastric ulcer in a rat model, and recorded the mesenteric afferent nerve firing and electrical properties of nodose ganglion neurons to explore the underlying mechanism. We provide evidence that SNP enhanced the spontaneous discharge of mesenteric afferent nerve, depolarized the membrane potential of NG neurons, and reduced the acetic acid-induced gastric ulcer. These effects were completely blocked by an S-nitrosylation blocking agent, reduced by a TRPV1 antagonist and genetic deletion. All these results indicate that NO might play a beneficial role in the gastric ulcer healing process via S-nitrosylation of TRPV1 on the vagus afferent fiber.

## Results

### SNP protected against acetic acid-induced gastric ulcer in rats via TRPV1 and vagus nerve

In order to investigate the protective effect of NO and if vagus nerve participates in this process of gastrointestinal inflammation, we tested the effect of sodium nitroprusside (SNP), a NO donor in an acetic acid-induced gastric ulcer model, with or without subdiaphragmatic vagotomy. Besides, abundant expression of TRPV1 was detected on vagal afferent neurons in the GI tract^23^. TRPV1 was also proved to be involved in the inflammatory response^24, 25^. To investigate if TRPV1 is involved in the ulcer protection effect of NO, a TRPV1 antagonist capsazepine (CZP) was also applied.

**Figure 1:**
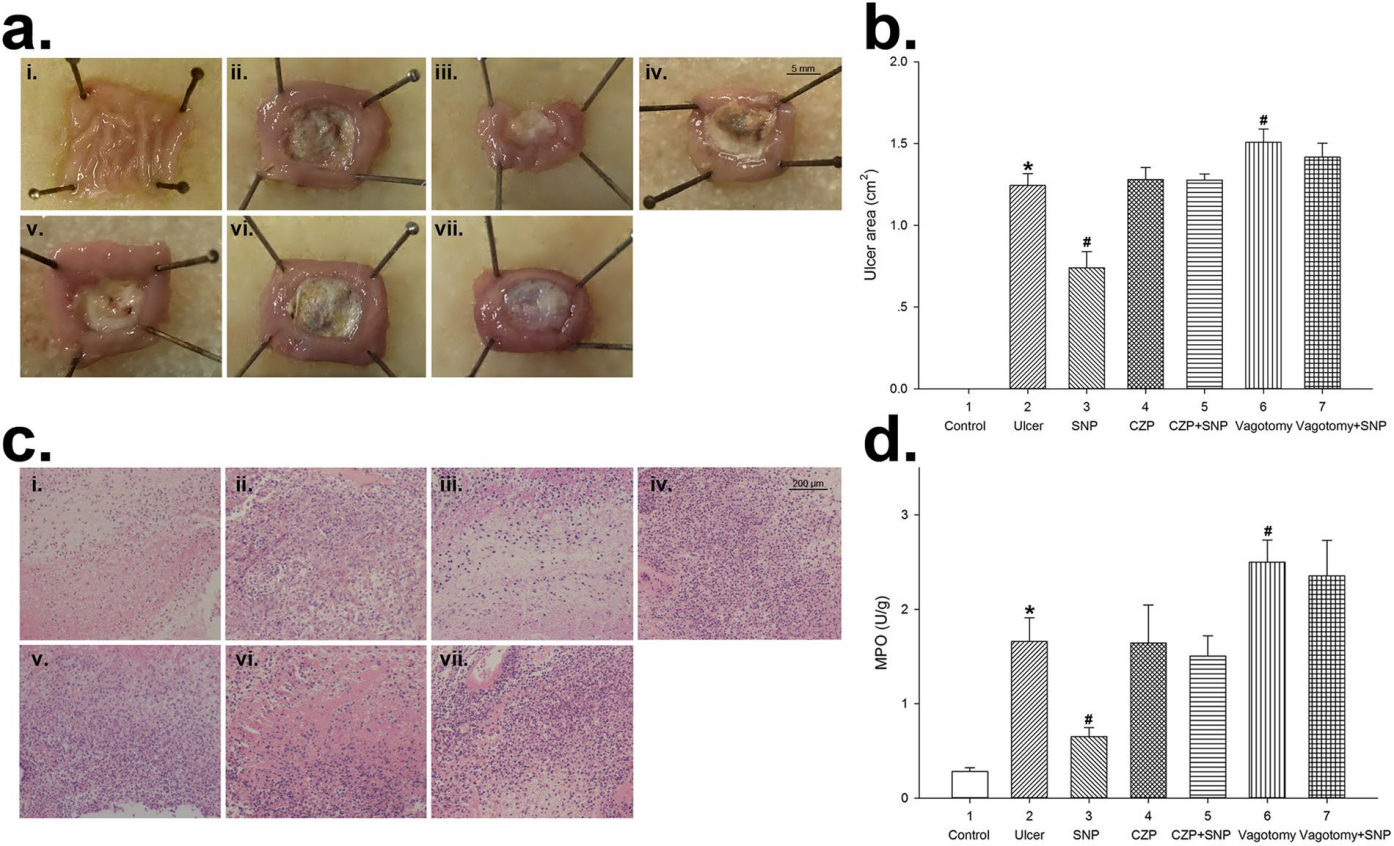
Sodium nitroprusside (SNP) alleviated acetic acid-induced gastric ulcer in rats, which was mediated by TRPV1 and vagus nerve. (**a**) Photographs showing the gross appearance of the gastric mucosa in rats treated with acetic acid in the wall of stomach compared with normal tissue of NS control group. Different sizes of ulcer are seen in the six acid-treated groups except the NS control. (i) NS control group; (ii) ulcer control group; (iii) SNP (3 mg/kg, i.p., bid) group; (iv) CZP (1.2 mg/kg, i.p., bid) group; (v) CZP + SNP group; (vi) vagotomy group; (vii) vagotomy + SNP group. (**b**) Summary values showing the gastric ulcer area (UA) in the seven groups. SNP treatment decreased UA. TRPV1 antagonist didn’t produce higher UA, but reduced the effect of SNP. Prior subdiaphragmatic vagotomy increased UA, and abolished the protective effective of SNP. *n* = 10 in vagotomy group, *n* = 6 in the other six groups, *P < 0.05 *vs* NS control group, ^#^ *P* < 0.05 *vs* ulcer control group, Dunn’s post hoc test after a one-way ANOVA. (**c**) Histopathological examination of the gastric mucosa in the five groups. (**d**) Summary values showing the local MPO levels in the rat stomach in each group. The MPO level of ulcer control group was significantly increased; that was decreased with the administration of SNP. Prior subdiaphragmatic vagotomy increased MPO activity. CZP or vagotomy abolished the effect of SNP. *n* = 8 in each column, *P < 0.05 *vs* NS control group, ^#^ *P* < 0.05 *vs* ulcer control group, Dunn’s post hoc test after a one-way ANOVA.

Four days after the administration of acetic acid, characteristic necrotic lesions were clearly produced in the anterior wall of the stomach, including loss of folds, edema, discoloration, white fur coating and mucosal necrosis in ulcer control group (Fig. 1aii). The extent of the ulcer area (UA) was 1.24 ± 0.07 mm^2^ (*n* = 6, Fig. 1b). Rats treated with SNP (3 mg/kg, i.p., bid) for four days exhibited considerably less inflammatory change and more intact fold around ulcer tissue. The UA was reduced to 0.74 ± 0.10 mm^2^ (*n* = 6, *P* < 0.05 *vs* ulcer control group, Fig. 1aiii and b). Pretreatment of TRPV1 antagonist didn’t produce severer inflammation, but reduced the protective effect of SNP on the ulcer (Fig. 1aiv,v). In SNP + CZP group, the UA was 1.28 ± 0.04 mm^2^ (*n* = 6), which was not significantly different from that of the CZP group (*n* = 6, *P* = 0.48, Fig. 1b). Subdiaphragmatic vagotomy exacerbated the mucous lesion caused by acetic acid. In the vagotomy group, the UA was 1.51 ± 0.08 mm^2^ (*n* = 10), larger than that of the ulcer control group (*n* = 6, *P* < 0.05, Fig. 1avi and b). The protective effective of SNP was abolished by vagotomy (UA, 1.42 ± 0.09 mm^2^, *n* = 6, *P* = 0.24 *vs* vagotomy group, Fig. 1avii and b).

The protective effect of SNP was confirmed histologically. Acetic acid induced severe damage of gastric mucosa, which was characterized by submucosa destruction, hemorrhagic injury and inflammatory cell infiltration (Fig. 1cii). Compared with the ulcer control rats, SNP-pretreated rats presented more intact histological structure of the mucosa. The mucosa destruction and leucocytes infiltration were much less severe. Pretreatment of CZP and vagotomy abolished this effect of SNP (Fig. 1ciii,v,vii).

On the 4th day after the treatment of acetic acid, the MPO activity in ulcer control group was increased to 1.66 ± 0.25 U/g (*n* = 8), higher than that of the NS control group (0.28 ± 0.04 U/g, *n* = 8, *P* < 0.05). The MPO activity was reduced in SNP group (0.65 ± 0.09 U/g, *n* = 8, *P* < 0.05 *vs* ulcer control group). Pretreatment of TRPV1 antagonist did not change the MPO activity in tissues (*n* = 8, *P* = 0.48 *vs* ulcer control group), but abolished the effect of SNP. The MPO activity was 1.50 ± 0.22 U/g in SNP + CZP group (*n* = 8), which was not significantly different from that of the CZP group (*n* = 8, *P* = 0.38, Fig. 1d). In the vagotomy group, the MPO activity was increased to 2.50 ± 0.23 U/g (*n* = 8, *P* < 0.05 *vs* ulcer control group). The MPO activity was 2.36 ± 0.37 U/g in vagotomy + SNP group (*n* = 8), which was not significantly different from that of the vagotomy group (*P* = 0.37, Fig. 1d).

### Effect of NO donor on spontaneous discharge of mesenteric afferent multi-unit activity in rats

**Figure 2:**
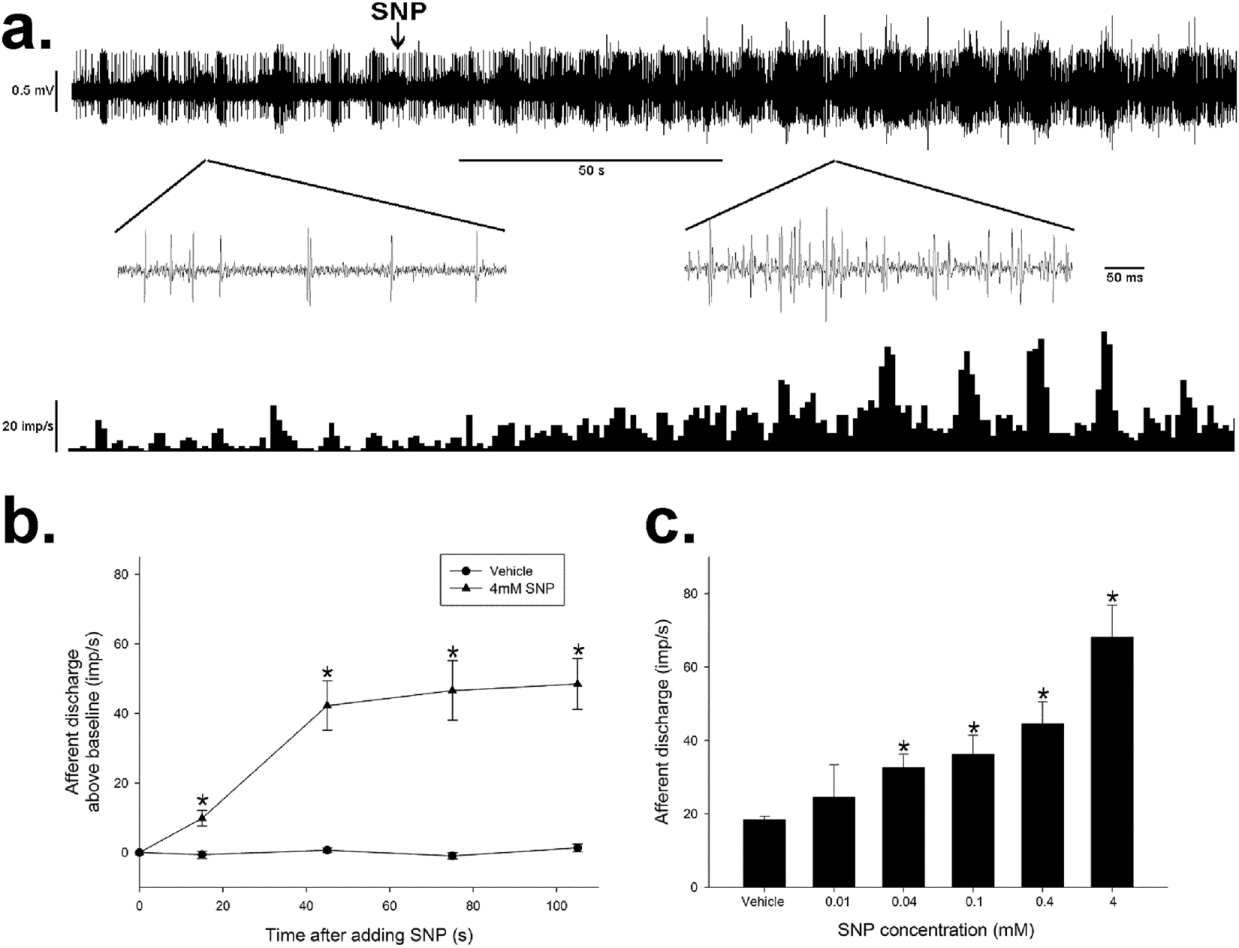
SNP increased the jejunal afferent spontaneous discharge in rats in a dose-dependent manner. (**a**) Raw recording of whole nerve activity before and after bath application of SNP (upper trace) and the number of nerve discharge in 3 s (lower trace). The inserts of the upper trace denote an expanded time-base to show the afferent spikes. The arrows indicate the application of SNP (4 mM) at the serosal side of the intestinal segment. (**b**) Time course of the excitatory effect of SNP (4 mM) on the spontaneous discharge rate of afferent nerve. The frequency of the afferent discharge increases at 20 s, reaches and maintains at plateau 50 s later. *n* = 6 in each group, *P < 0.05 *vs* vehicle, Students’ *t*-test. (**c**) Dose-response character for the excitatory effect of SNP (0.01–4 mM) on spontaneous discharge rate. The data shown in the columns are the frequencies of afferent discharge at 105 s following SNP or vehicle administration. *n* = 6 in each group, *P < 0.05 *vs* vehicle, Students’ *t*-test.

To testify the hypothesis that NO might initiate the vagal anti-inflammatory reflex in GI tract, we examined the actions of SNP on spontaneous mesenteric afferent discharge. When added to the Krebs perfusion at the serosal side of the gut segment for 2 min, SNP elicited significant increase in afferent multiunit firing rates (Fig. 2a). Following SNP (4 mM) administration, the frequency of afferent spikes began to increase at 25.4 ± 0.4 s, reached the plateau at 73.1 ± 6.8 s, and maintained at that level even after flushing the perfusion fluid for 15 min. The mean discharge above baseline at 105 s was increased from 1.3 ± 1.2 imp/s (vehicle control) to 48.5 ± 7.3 imp/s (SNP 4 mM, *P* < 0.05, *n* = 6, Fig. 2b). The afferent nerve activation effect of SNP acted in a dose-dependent manner, and the half-maximum effective dose (ED_50_) was 0.16 mM (*n* = 6 in each column, Fig. 2c).

### Effect of NO donor on spontaneous discharge of different subunits of mesenteric afferent fibers

**Figure 3:**
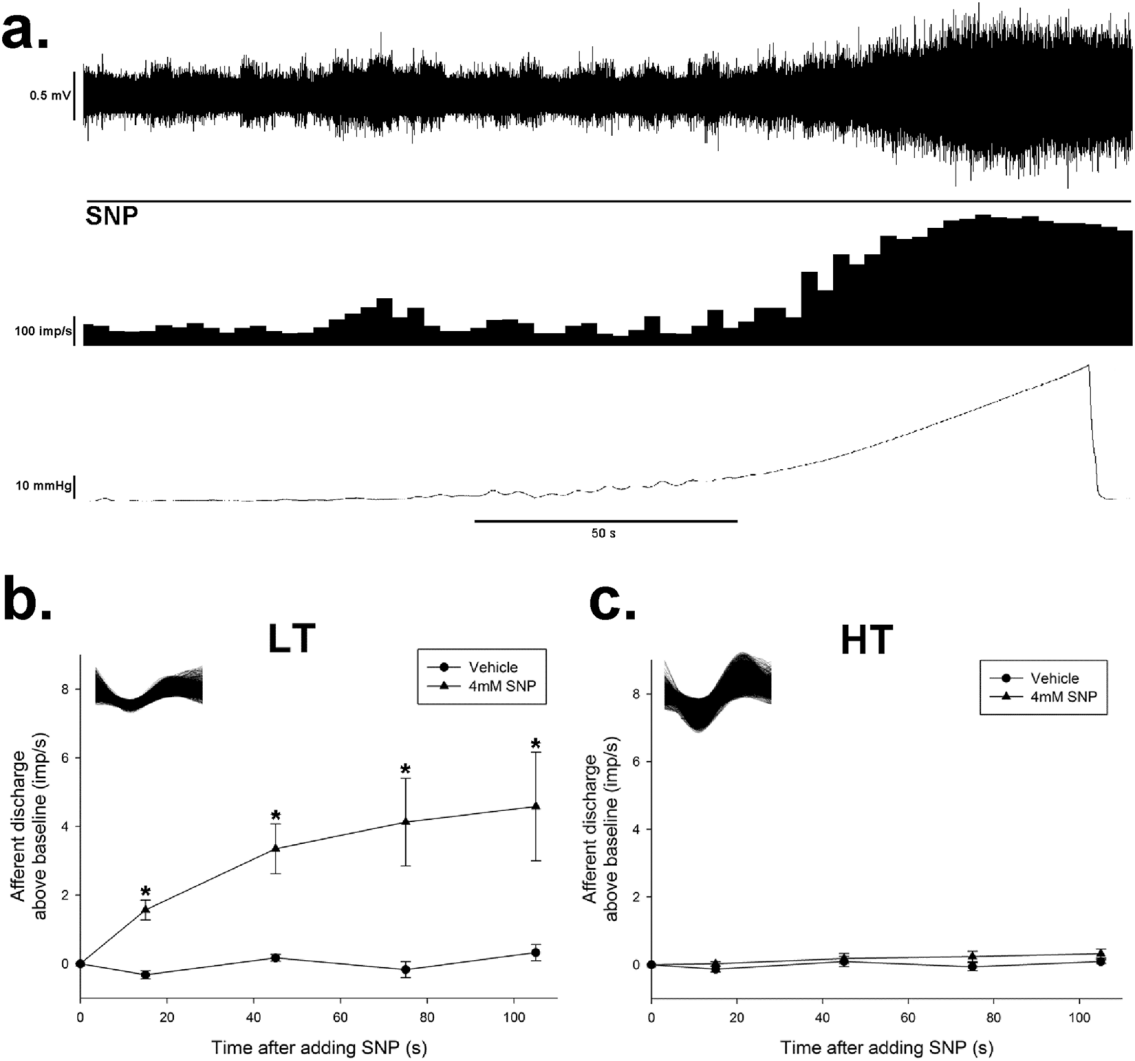
Effect of SNP on different subtypes of afferent nerve. (**a**) Responses of the multiunit mesenteric afferent fibers to ramp jejunal distension. The upper trace shows the raw recording of whole nerve discharge in the presence of SNP (4 mM), the middle trace is the number of nerve discharge in 3 s, and the lower trace shows corresponding intraluminal pressure. The multiunit discharge was divided into low threshold (LT) unit, high threshold (HT) unit, wide dynamic range (WDR) unit and mechanically insensitive afferents (MIA). The effect of SNP on the LT and HT units are shown in (**b**,**c**). (**b**) Time course of the excitatory effect of SNP (4 mM) on the spontaneous discharge rate of low threshold units. Top left corner shows identifiable shapes of low threshold units. *n* = 6 in each group, *P < 0.05 *vs* vehicle, Students’ *t*-test. (**c**) Time course shows the frequency of spontaneous firings of high threshold single units were not altered by adding SNP. Top left corner shows superimposed traces identifying high threshold units. *n* = 6 in each group, Students’ *t*-test.

To identify the subtype of the mesenteric afferent nerve activated by SNP, the mechanical sensitivity of the spontaneous afferent spikes was assessed by ramp distension 5 min after SNP (4 mM) administration. The jejunal afferent firing increased in a pressure-dependent manner. Based on their spike waveform, 62 single units were discriminated in the multiunit recordings of 5 rats. According to their response to distension, 17 units was identified as the low thresholds (LT, maximal discharge rates at <20 mmHg), 22 as the wide dynamic range (WDR, increased monotonically over the entire range of 60 mmHg), 20 as the high thresholds (HT, activation threshold >20 mmHg), and 3 as mechanically insensitive afferents (MIA, unresponsive to ramp distension). SNP activated the spontaneous afferent firing of LT units (*P* < 0.05 *vs* vehicle, Fig. 3b), while did not influence that of the HT units (Fig. 3c).The mesenteric afferent nerve bundle contains both vagal and spinal fibers, and most of LT are vagus afferent fiber, while most of HT are spinal afferent fibers^26^. So it is possible that NO in GI tract selectively excite the afferent fiber. These data support our hypothesis that NO in GI tract might excite the vagal anti-inflammation reflex.

### Vagotomy eliminated the excitatory effects of SNP on spontaneous afferent discharge

**Figure 4:**
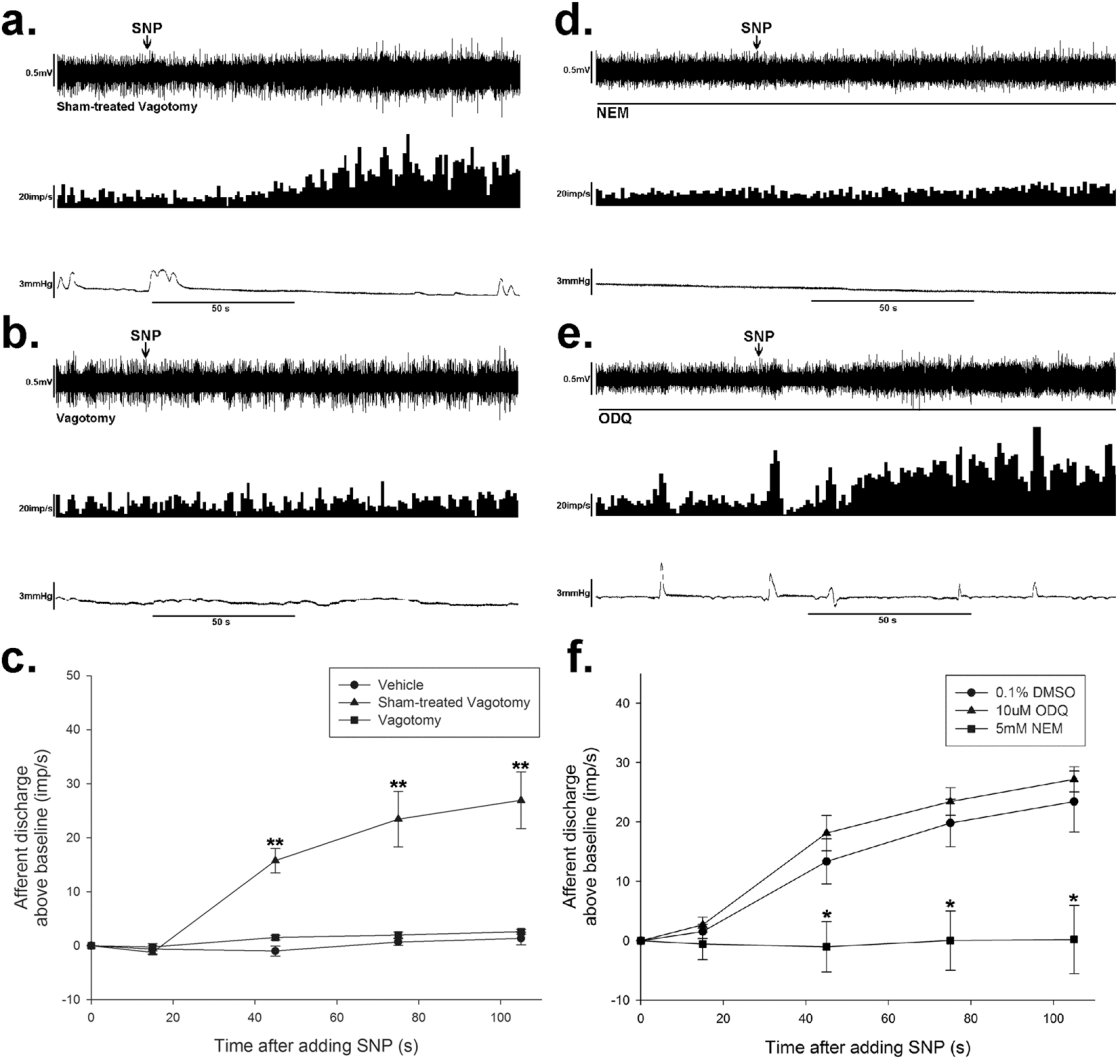
The excitatory effect of SNP was abolished by prior subdiaphragmatic vagotomy and S-nitrosylation blocker NEM. (**a**) Response of whole nerve activity before and after SNP administration on sham-operation rats. The upper trace shows the raw recording of whole nerve discharge, the middle trace is the number of nerve discharge in 3 s, and the lower trace shows corresponding intraluminal pressure. The arrows indicate the administration of SNP (0.16 mM). Sham-operation of vagotomy didn’t change the effect of SNP to increase the spontaneous afferent discharge. (**b**) Response of whole nerve activity to SNP on subdiaphragmatic vagotomy rats. SNP failed to increase the spontaneous afferent discharge. (**c**) Summary values showing that the excitatory effect of SNP (0.16 mM) was eliminated by vagotomy. *n* = 6 in each group, **P < 0.01 *vs* vehicle, Dunn’s post hoc test after a one-way ANOVA. (**d**) Response of whole nerve activity to SNP in the presence of NEM (5 mM). SNP (0.16 mM) failed to increase the spontaneous afferent discharge. (**e**) Response of whole nerve activity to SNP in the existence of ODQ (10 µM). ODQ didn’t change the excitatory effect of SNP on the spontaneous afferent discharge. (**f**) Summary values showing that the excitatory effect of SNP (0.16 mM) was eliminated by NEM (5 mM), while ODQ (10 µM) had no effect compared to the DMSO group. *n* = 6 in each group, *P < 0.05 *vs* DMSO group, Dunn’s post hoc test after a one-way ANOVA.

To further test our hypothesis that SNP could selectively excite the vagus afferent fiber, prior subdiaphragmatic vagotomy was performed. CCK was used as a control to conform that surgical vagotomy quenched all the vagal nerve due to its overt activation of vagal but not spinal afferent^27^. After vagotomy, the afferent activation induced by CCK (100 pM) was completely eliminated (see Supplementary Fig. S1). In vagotomy group, the administration of SNP did not influence the spontaneous discharge of mesenteric afferent nerve discharge (*n* = 6, *P* = 0.30 *vs* vehicle, Fig. 4b). The mean discharge above baseline was decreased from 26.9 ± 5.2 imp/s (sham-operation + SNP group) to 2.6 ± 0.4 imp/s (vagotomy + SNP group, *P* < 0.01, *n* = 6, Fig. 4c).

### Involvement of S-nitrosylation and TRPV1 in the SNP-induced activation of the mesenteric afferent nerve

To further investigate whether SNP activates mesenteric afferent nerve via the NO/sGC/cGMP/PKG signaling pathway, or through S-nitrosylation of target proteins, pharmacological experiments were performed using 1H-[1,2,4] oxadiazolo [4,3-a] quinoxalin-1-one (ODQ), a selective inhibitor of guanylate cyclase, and N-ethylmaleimide (NEM), a protein thiol protective agent. Pretreatment with NEM for 15 min completely eliminated the excitatory effect of SNP on the mesenteric afferent nerve (Fig. 4d). The mean discharge above baseline was decreased from 23.4 ± 5.2 imp/s (DMSO +SNP group, SNP 0.16 mM) to 0.2 ± 5.7 imp/s (NEM + SNP group, NEM 5 mM, *P* < 0.05, *n* = 6, Fig. 4f). However, the presence of ODQ (10 µM, 10 min) did not exert any influence on the excitatory effect of SNP (*P* = 0.52 *vs* DMSO control, *n* = 6, Fig. 4e). These findings suggest that the effect of SNP might be mediated via S-nitrosylation.

**Figure 5:**
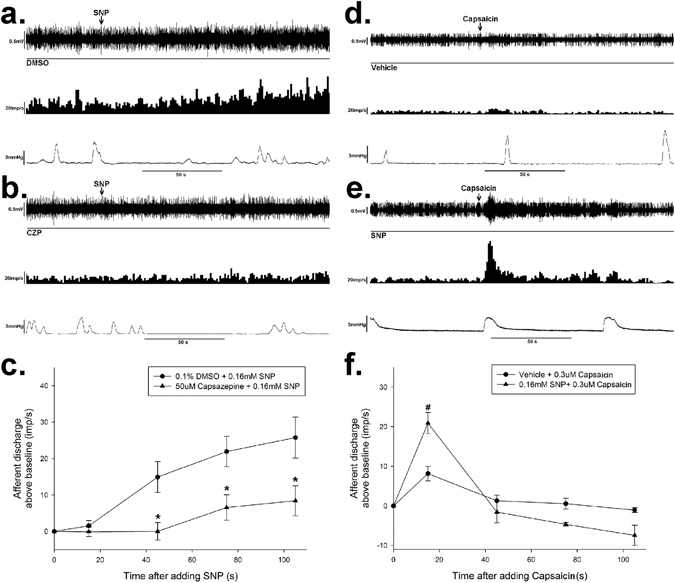
The excitatory effect of SNP was mediated by TRPV1. (**a**) Response of whole nerve activity before and after SNP administration in the presence DMSO (0.1%). The upper trace shows the raw recording of whole nerve discharge, the middle trace is the number of nerve discharge in 3 s, and the lower trace shows corresponding intraluminal pressure. The arrows indicate the administration of SNP (0.16 mM). DMSO didn’t change the excitatory effect of SNP on the spontaneous afferent discharge. (**b**) Response of whole nerve activity to SNP in the existence of CZP (50 µM). SNP (0.16 mM) failed to increase the spontaneous afferent discharge. (**c**) Summary values showing that the excitatory effect of SNP (0.16 mM) was inhibited by CZP (50 µM). *n* = 6 in each group, *P < 0.05 *vs* DMSO group, Students’ *t*-test. (**d**) Response of whole nerve activity to capsaicin (0.3 µM). The application of capsaicin increased the spontaneous afferent discharge. (**e**) Response of whole nerve activity to capsaicin (0.3 µM) in the presence of SNP (0.16 mM). SNP markedly increased the capsaicin-evoked mesenteric afferent firing. (**f**) Summary values showing that the excitatory effect of capsaicin (0.3 µM) was enhanced by SNP (0.16 mM). *n* = 6 in each group, ^#^ *P* < 0.01 *vs* vehicle, Students’ *t*-test.

To investigate if TRPV1 is involve in the excitatory effect of SNP, TRPV1 antagonist CZP was also applied on the serosal side of the gut segment. Pre-treatment of CZP (50 µM) completely diminished the excitatory effect of SNP on mesenteric afferent discharge. The mean frequency of the afferent discharge was decreased from 25.8 ± 5.6 imp/s (DMSO +SNP group, *n* = 6) to 8.4 ± 4.1 imp/s (CZP + SNP group, *n* = 6, *P* < 0.05, Fig. 5c). SNP also markedly enhanced the excitatory effect of capsaicin (0.3 µM), the agonist of TRPV1, on the mesenteric afferent discharge. The mean afferent discharge was increased from 8.2 ± 1.8 imp/s (capsaicin control group) to 20.9 ± 2.7 imp/s (SNP 0.16 mM, *P* < 0.05 *vs* capsaicin control group, *n* = 6, Fig. 5f).

**Figure 6:**
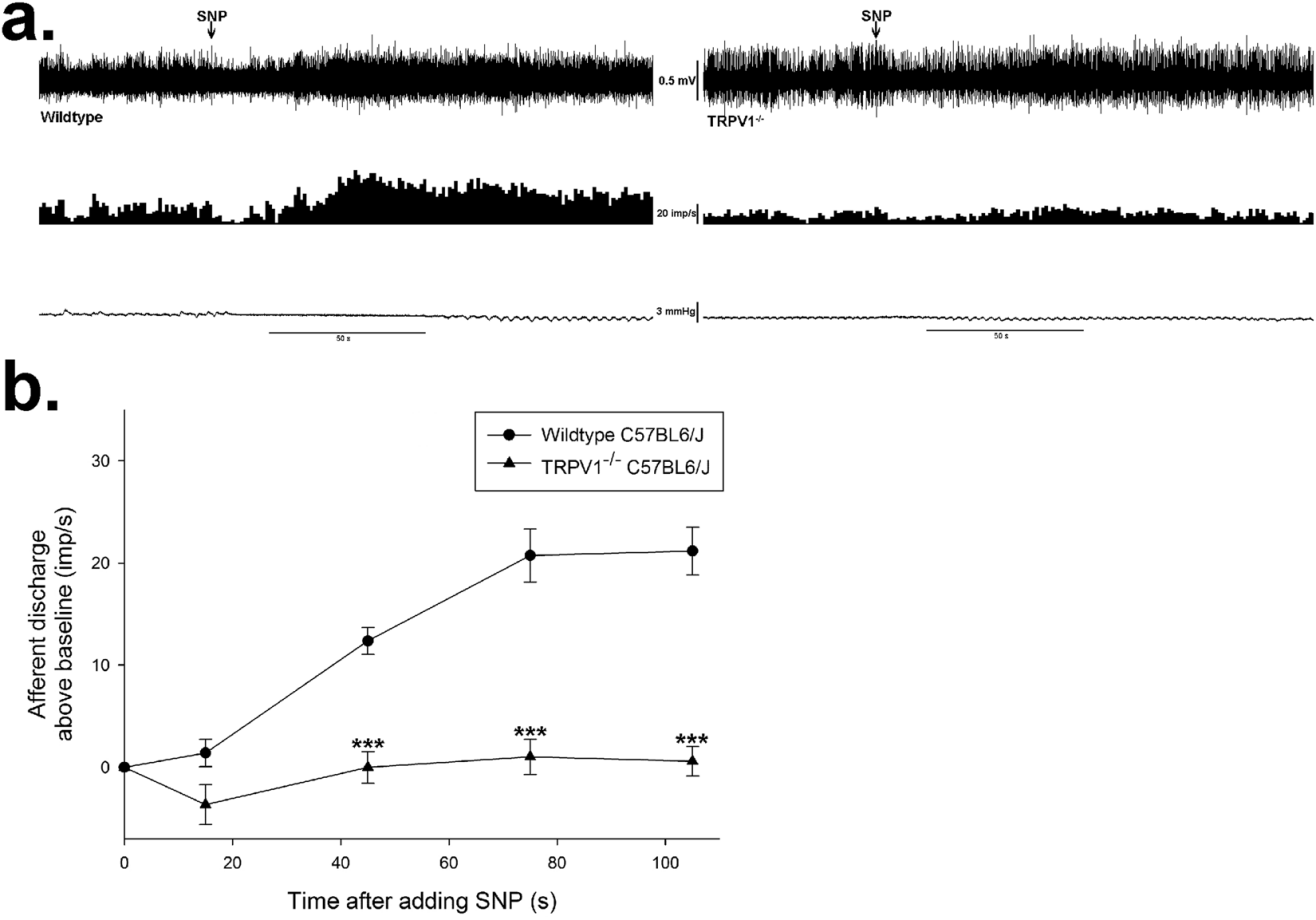
The excitatory effect of SNP was abolished in TRPV1^−/−^ mice. (**a**) Representative spontaneous afferent discharge recordings of response to SNP (0.16 mM) on mice. The upper trace shows the raw recording of whole nerve discharge, the middle trace is the number of nerve discharge in 3 s, and the lower trace shows corresponding intraluminal pressure. The arrows indicate the administration of SNP (0.16 mM). SNP increased the afferent discharge of wild type C57BL6/J mice, but failed to increase the nerve firing of TRPV1^−/−^ mice. (**b**) Summary values showing that the TRPV1 genetic deletion eliminated the excitatory effect of SNP (0.16 mM). *n* = 6 in each group, ***P < 0.001 *vs* wild type C57BL6/J group, Students’ *t*-test.

To further confirm these results, we explored the effect of SNP on mesenteric afferent nerve discharge from TRPV1-deficient (TRPV1^−/−^) mice. Administration of SNP elicited similar activation towards afferent discharge of wild type C57BL6/J mice, while failed to increase the nerve firing of TRPV1^−/−^ mice (0.6 ± 1.4 imp/s *vs* 21.2 ± 2.3 imp/s, *n* = 6 in each group, *P* < 0.001, Fig. 6).

### Effect of NO donor on resting membrane potential and capsaicin-induced inward current of NG neurons of rats by whole-cell clamp patch

**Figure 7:**
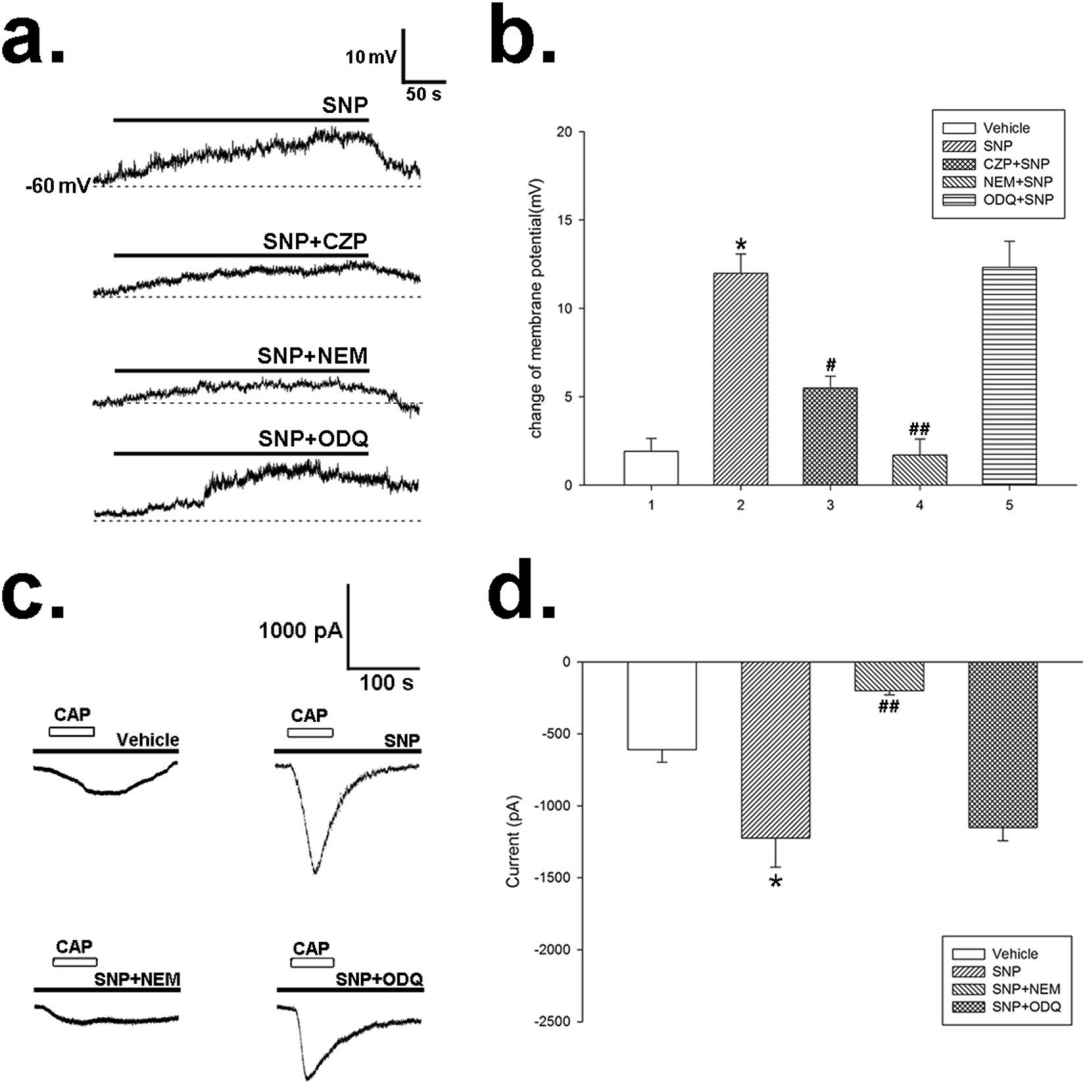
The effect of SNP on membrane potential and capsaicin-induced inward current (*Icap*) in cultured NG neurons. (**a**) Representative membrane potential traces of the NG neurons after administration of SNP (0.16 mM), or co-application of SNP and NEM (5 mM) or ODQ (10 µM). The filled bars on the trace represent the administration of corresponding drugs. (**b**) Summary values show that application of SNP (0.16 mM) depolarized the membrane potential of NG neurons gradually. CZP (50 µM) or NEM (5 mM) blocked the effect of SNP, while ODQ (10 µM) had no effect. *n* = 7 in each column, *P < 0.05 *vs* vehicle, ^#^ *P* < 0.05 *vs* SNP group, ^##^ *P* < 0.01 *vs* SNP group, Dunn’s post hoc test after a one-way ANOVA. (**c**) Representative traces of *Icap* after incubation of SNP (0.16 mM), or co-application of SNP and NEM (5 mM) or ODQ (10 µM). NEM eliminated the activation of *Icap* caused by SNP. Open bars on the trace represent the application of capsaicin for 60 s, and the filled bars indicate the pretreatment of corresponding drugs. (**d**) Summary values show that administration of SNP (0.16 mM) increased *Icap*. This effect was abolished by NEM, but not ODQ. *n* = 7 in each column, *P < 0.05 *vs* vehicle, ^##^ *P* < 0.01 *vs* SNP group, Dunn’s post hoc test after a one-way ANOVA.

In order to testify the hypothesis that NO modulates the vagal afferent discharge, we recorded the membrane potential and current of the neurons in NG in rats by whole-cell clamp patch. The resting membrane potential of NG neurons was −57.4 ± 1.3 mV (*n* = 43). The membrane was depolarized following SNP (0.16 mM) administration, and partially recovered after washing the cells with extracellular fluid (Fig. 7a). Pretreatment of CZP (50 µM) or NEM (5 mM) significantly diminished this effect, while ODQ did not influence it (Fig. 7b). These results were consistent with that on the mesenteric afferent recording.

To further determine whether TRPV1 is the key target of NO S-nitrosylation, we examined the effect of SNP on capsaicin-induced inward current (*Icap*) on cultured NG neurons. Shortly after capsaicin (1 μM) applied in the bath, an inward current was recorded on the membrane of NG neurons (Fig. 7c). Since *Icap* could be decreased by repetitive applications of capsaicin^28^, we applied capsaicin only once on every recorded NG neuron. Pretreatment of SNP (0.16 mM) increased the amplitude of *Icap* significantly, from −609.4 ± 88.0 pA to −1224.3 ± 202.1 pA (*P* < 0.05, *n* = 7). This effect of SNP was abolished by NEM (5 mM), but was not influenced by ODQ (10 µM) (Fig. 7d).

## Discussion

This study demonstrated the role of NO donor SNP to protect against gastric ulcer in rats, which was diminished by TRPV1 antagonist or vagotomy. SNP increased the jejunal mesenteric afferent discharge, depolarized the resting membrane potential of NG neurons, and enhanced capsaicin-induced inward current. S-nitrosylation of TRPV1 might be involved in these processes.

As one of the most studied signaling molecules, the role of NO is currently obscure in GI physiology and pathophysiology. The administration of NO-donors plays a positive role in healing of gastric mucosa damage and experimental gastric ulcers^29^. NO donor GTN demonstrates gastro-protective properties against ethanol^30^. Another donor SNAP attenuates ethanol-induced gastric lesions^31^. The treatment of Lactobacillus farciminis to produce NO *in vitro* attenuates the colonic damage in TNBS-induced colitis, and NO donor SNP exerts similar effect^32^. The gastro-protection effect of NO donors is attributed to the increase of gastric blood flow, the inhibition of gastric acid secretion, and the maintenance of gastric mucosal barrier integrity^33, 34^. During gastrointestinal inflammation, cytokines and endotoxins activate an anti-inflammatory loop to the gut^17, 35^. The anatomical evidence indicates that vagus nerve is involved in this loop^36^. So, it is recognized that vagus nerve plays an important role for the maintenance of the immunological homeostasis in the intestine. Most work to date has focused on the cholinergic efferent branch of this reflex, but little is known about the afferent mechanism.

In the present research, we found that peritoneal injection of SNP inhibited the acetic acid-induced gastric ulcer in rats, and this effect was abolished by vagotomy. So, for the first time we provided evidence that vagus nerve was involved in the protective effect of NO on the gastrointestinal ulcer. In other words, besides other mechanisms, NO might exert its protective effect on the GI tract via vagus nerve. The underlying mechanism might be excitation of the vagal anti-inflammatory reflex.

Initially recognized as endothelium-derived relaxing factor (EDRF)^37^, NO has been shown to have multiple functions in numerous tissues and cells^38^. The effect of NO in peripheral nerve system is still far from settled. NO modulates articular C-fiber activity and reduces its responsiveness to bradykinin^39^. Exogenous NO has dual effect on mechanosensitivity^40, 41^. NO donor triggers vagal afferent activation in rat stomach^20^, and decreases soma excitability of vagal sensory neurons^42^.

In our present research, we used jejunal mesenteric afferents recording to test the effect of exogenous NO on the visceral sensory fibers. The data indicated that serosal administration of SNP dose-dependently increased the spontaneous discharge of afferents fibers. Besides this, we also found that SNP excited the LT units, which was considered to be vagal afferent fiber, but did not influence that of HT, most of which were spinal afferent fiber^26^. Subdiaphragmatic vagotomy absolutely diminished the effect of SNP. SNP also depolarized the resting membrane potential of NG neurons in a dose-dependent manner. Thus, we believed that exogenous NO might selectively activate vagus afferents.

S-nitrosylation is a reversible covalent bonding of a nitrogen monoxide group to the thiols of protein. This post-translational modification requires higher concentration of NO and tends to proceed with slower kinetics than the traditional cGMP pathway^11^. S-nitrosylation plays a central role in NO-mediated cellular signal transduction^10, 43^. Most classes of proteins and ion channels have been identified as the targets for S-nitrosylation^8, 44^, including TRPV1^25^.

Our study proved that N-ethylmaleimide (NEM), which covalently prevented thiol groups from nitrosylation, completely abolished the excitatory effect of SNP, while ODQ, a selective inhibitor of guanylate cyclase, had no effect. NEM also blocked the effect of SNP in depolarizing the membrane potential of cultured NG neurons. This was a proof that NO donor activated mesenteric afferents and NG neurons via S-nitrosylation. The frequency of afferent spikes maintained at a high level even after flushing the perfusion fluid was due to the long-term effect of S-nitrosylation.

As a nonselective cation channel that detects a wide range of stimuli such as capsaicin, heat, and acid^45–47^, TRPV1 displays remarkable gating diversity and plays a pivotal role in thermal nociception and inflammatory hyperalgesia^48–50^. TRPV1 deletion increases local inflammation and enhances systemic inflammatory response^24^. Syndrome NO donor SNAP enhances the sensitivity of TRPV1 to H^+^ despite normal surface expression^25^. NO donor SNP induces apoptosis which is associated with TRPV1 channel-mediated Ca^2+^ entry via S-nitrosylation in osteoblasts^51^. Spinal and vagal primary afferent neurons are the major cellular expression sites of TRPV1 in the GI tract^23^. About 70% of the afferent vagal fibers express TRPV1^52^, and capsaicin activates approximately 30% of GI vagal afferents^53^. Accumulated evidences support that TRPV1 makes an important contribution to gastrointestinal sensory transduction and inflammatory nociception. But there is no direct evidence to prove that TRPV1 participates in the vagal anti-inflammatory loop.

Our pharmacological studies revealed that TRPV1 might act as a key point of SNP protection, and also participated in the SNP-induced activation of jejunal mesenteric afferents and NG neurons. SNP significantly increased the mesenteric afferent sensitivity to capsaicin, and capsaicin-induced inward current of NG neurons. Pre-treatment of CZP and TRPV1 knock-out decreasd the effect of SNP. NEM pretreatment blocked the excitatory effect of SNP on capsaicin-induced inward current.

In conclusion, we demonstrate that exogenous NO may participate in the vagal inflammatory reflex to improve gastric ulcer healing via TRPV1 S-nitrosylation. This study will provide new data about the afferent pathway of the intestinal inflammation reflex, and provide a potential target for the pharmacological treatment of gastrointestinal inflammatory disease.

## Methods

### Experimental animals

Male Wistar rats (200–270 g) and wild type C57BL6/J mice (6–8 weeks) were procured from the Animal Center of Shandong University. Male TRPV1-dificient mice (6–8 weeks) were kindly provided by Dr. Yu Xiao (Shandong University). The investigation was in accordance with the guide for the care and use of laboratory animals published by the U.S. National Institutes of Health (NIH Publication No. 85–23). All experiments were approved by the Medical Ethics Committee for Experimental Animals, Shandong University School of Basic Medical Sciences (ECAESDUSM2014056).

### Induction of Gastric ulcer

All rats were fasted for 24 hours before surgery. After the animals were anaesthetized with 2% sodium pentobarbital (60 mg/kg), an upper midline laparotomy was carried out. The lobes of the liver were gently pulled aside and the anterior wall of the stomach was exposed. After the injection of 30 μL acetic acid or normal saline into the submucosa near the antrum, the wound was covered with omentum majus to avoid acid leaking. The peritoneal cavity was sprinkled with antibiotics to prevent infection, and then abdominal muscle and skin were sutured.

The animals were randomly divided into 7 groups, including: NS control group, ulcer control group, SNP (3 mg/kg, i.p., bid) group, CZP (1.2 mg/kg, i.p., bid) group, CZP + SNP group, vagotomy (14 days before the experiment) group, vagotomy + SNP group. Two control groups and vagotomy group were given normal saline (3 ml/kg, i.p., bid). The gastric ulcer area reached the peak at 3^rd^ to 4^th^ day after the treatment of acetic acid, and began to heal thereafter^54^. So, in this study, we sacrificed the rats by cervical dislocation at the 4^th^ day. The stomachs were opened by the greater curvature, and rinsed with Krebs solution to take photographs of the mucosal surface. The extent of the ulcer areas in the five groups were measured (UA, mm^2^). The gastric lesions were hemisected. One half of it was immediately fixed in 10% formalin for 24 h, and then dehydrated in 100% ethanol, cleared in xylene and embedded in paraffin. The other half was stored at −80 °C for MPO analysis. The histologic samples were cut into 4 μm-thick sections and stained with hematoxylin-eosin (HE, NanJing Jiancheng Bioengineering Institute, Nanjing, China).

### Vagotomy

A subdiaphragmatic vagotomy was performed. All rats were fasted for 24 hours before surgery. After anesthesia, an upper midline laparotomy was performed. Subdiaphragmatic vagal trunks (both the ventral and dorsal branches) were carefully identified and transected. In sham-operation animals, vagal branches were visualized but not amputated. After surgical sutures, rats were raised to recover for 14 days before electrophysiological experiments. To assess the completeness of the vagotomy, all vagotomized animals were tested by recording the jejunal afferent response to cholecystokinin (CCK; AnaSpec, Fremont, CA). Only rats exhibiting no response to CCK were considered successful vagotomy^27, 55, 56^.

### Measurement of myeloperoxidase activity

The inflammatory degree of the gastric lesions was also measured by myeloperoxidase (MPO) activity, which represented neutrophil infiltration in the damaged tissue. MPO activities of the tissue samples homogenates in 1:19 (w/v) buffer solution in each group were measured using MPO detection kits (Jiancheng Bioengineering Institute, Nanjing, China) by a spectrophotometer at 460 nm. One unit of MPO activity was defined as the quantity of enzyme that decomposed 1 μmol H_2_O_2_ per min at 37 °C in 1 g of wet tissue.

### Recording of jejunal afferent nerve activity

Jejunal afferent nerve recording was carried out as previously described^57^. In brief, a midline laparotomy was performed in rats/mice after anesthesia. Segment of jejunum (2 cm) with attached mesentery was quickly cut off, the lumen contents were cleaned away, and the emptied segment was transferred to an organ bath with continuously Krebs buffer perfusion (in mM: NaCl 118, KCl4.8, NaHCO_3_25, NaH_2_PO_4_1.0, MgSO_4_1.2, glucose11.1, CaCl_2_ 2.5, gassed with 95% O_2_ - 5% CO_2_, 34 °C, rate 10 mL/min). The oral end of the lumen was attached to an input port perfused with Krebs solution (rate 10 mL/h), while the anal end was open. Under microscope, the nerve bundle was gently isolated and attached to a recording electrode. Nerve activities were input to a single channel 1902 preamplifier/filter (Cambridge Electronic Design, Cambridge, UK), amplified (10000 times), filtered (band pass 100–1000 Hz), passed to Micro 1401 interface system (CED) and viewed on a personal computer running Spike 2 software (version 5.01; CED).

Before any experimental procedures, the nerve activity was recorded for 10 min to stabilize. Ramp distension was produced by temporarily closing the outlet port and increasing the intraluminal perfusion speed to 30 mL/h. This allowed the segment to be distended to an intraluminal pressure up to 60 mmHg.

### Culture of neurons from nodose ganglion

Both the left and right NG neurons were quickly excised from cervical dislocation-killed rats. After desheathed in ice-cold sterile Krebs solution, the ganglia were incubated for 50 min in digestion buffer of collagenase type I (1 mg/mL, Sigma) and trypsin II-S (0.5 mg/mL, Sigma) in 5 mL DMEM medium (Gibco, Gaithersburg, MD, USA) at 37 °C. The enzymatic reaction was terminated by soybean trypsin inhibitor type II-S (0.625 mg/mL, Sigma). The ganglia were then transferred to 1 mL DMEM tissue culture medium containing 10% fetal bovine serum (Gibco). A single-cell suspension was created by repeated mechanical trituration using a plastic Pasteur pipe. The NG neurons were plated onto poly-l-lysine-coated dishes and then incubated at 37 °C in a 5% CO_2_ incubator (Thermo Forma, Hamilton, NJ, USA).

### Whole-cell patch-clamp recording

Whole-cell patch-clamp recordings were carried out by an Axon Instruments Multiclamp 700B amplifier (Molecular Devices, New York, NY, USA) interfaced to Digidata 1440 A with the pClamp 10.2 software (Molecular Devices). Patch pipettes of 5–7 MΩ resistance in extracellular fluid were used to contact cells.

### Data analysis

For jejunal afferent recording, a time constant of 2 min recording before administration of any drugs or vehicle was taken as the baseline firing frequency (impulse/s). Responses to stimulations were evaluated by the average impulse frequency above baseline every 30 s up to 120 s following the drug administration. Single unit analysis was performed by the template matching algorithm in SPIKE 2 (CED) as described previously^58^.

For patch-clamp recording, the baseline was determined by the change of membrane potential after the treatment of extracellular fluid.

### Statistics

All statistical analyses were performed by Sigmaplot statistical software (Sigma). All data are presented as mean ± SEM. Data were evaluated using Students *t*-test, or one-way ANOVA followed by post hoc Dunnett’s multiple comparison tests. The number of animals or neuron cells was indicated as *n* and *P* < 0.05 was considered to be statistically significant.

### Chemicals

CCK was obtained from AnaSpec (Fremont, CA), DMEM and fetal bovine serum was obtained from Gibco (Gaithersburg, MD, USA), other drug compounds were purchased from Sigma-Aldrich Corp (St. Louis, MO, USA). Capsaicin, CZP, NEM, ODQ and CCK were dissolved in dimethyl sulphoxide (DMSO) to make stock solutions. On the day of the experiment the aliquots were diluted in Krebs to working concentrations with final DMSO concentrations of ≤0.1%.

## Electronic supplementary material

Supplementary Information
