# Supplementary material for: Nitric oxide donor protects against acetic acid-induced gastric ulcer in rats via S-nitrosylation of TRPV1 on vagus nerve
Source: Sci Rep. 2017 May 18;7:2063. doi: 10.1038/s41598-017-02275-1 (PMC5437002; doi:10.1038/s41598-017-02275-1)

Nitric oxide donor protects against acetic acid-induced gastric ulcer in rats via S-nitrosylation of TRPV1 on vagus nerve

Ting Han[1], Yan Tang[1], Jing Li[1], Bing Xue[1], Liping Gong[1], Jingxin Li[1], Xiao Yu[1]& Chuanyong Liu[1,2]

[1]Department of Physiology, School of Basic Medical Sciences, Shandong University Cheeloo Medical College, China.

[2]Provincial Key Lab of Mental Disorder, School of Basic Medical Sciences, Shandong University Cheeloo Medical College, China.

Correspondence to:

Chuanyong Liu, Department of Physiology, School of Basic Medical Sciences, Shandong University Cheeloo Medical College, China.

Tel: +86-531-88380018; fax: +86-531-88383902.

E-mail address: liucy@sdu.edu.cn.

Supplementary Figure S1. The excitatory effect of CCK on mesenteric afferent discharge was abolished by prior subdiaphragmatic vagotomy.

(a) Response of whole nerve activity before and after CCK (100 pM) administration on sham-operation and vagotomy rats. The upper trace shows the raw recording of whole nerve discharge, the middle trace is the number of nerve discharge in 3 sec, and the lower trace shows corresponding intraluminal pressure. The arrows indicate the administration of CCK (100 pM). After vagotomy, CCK failed to increase the spontaneous afferent discharge.

(b) Summary values showing that the excitatory effect of CCK (100pM) was eliminated by vagotomy. n = 6 in each group, * $P < 0.05$ *vs* sham-operation group, Students' t-test.

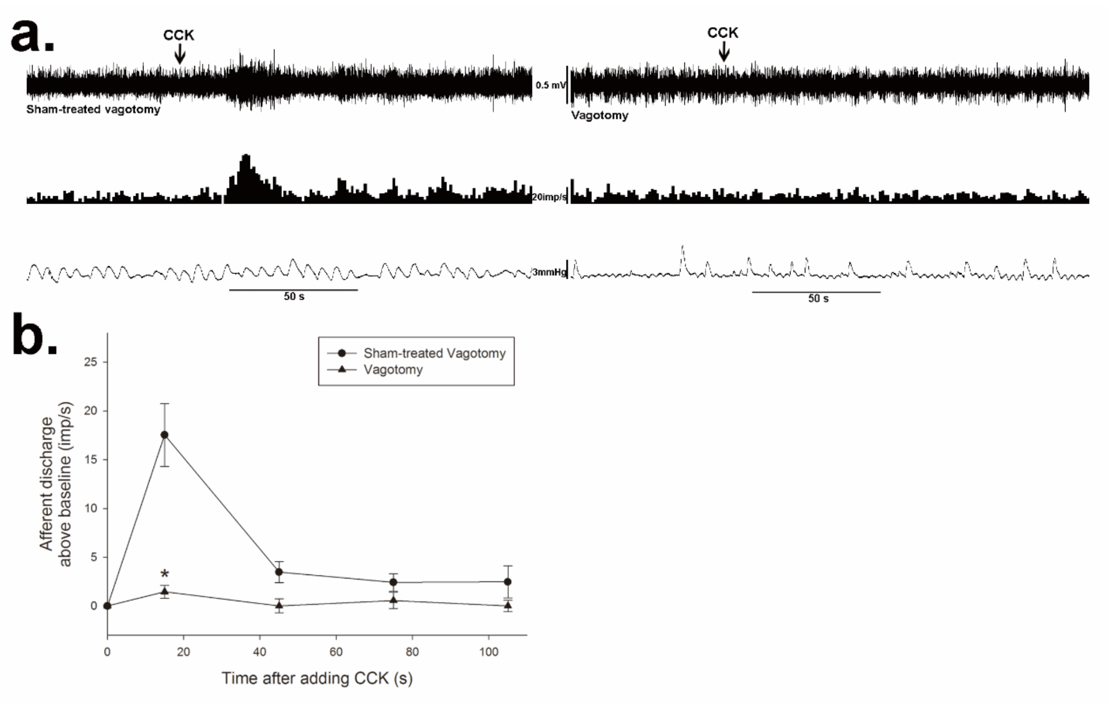

Supplement: Supplementary file 1 — Supplementary Information [file 41598_2017_2275_MOESM1_ESM.pdf]
